# Annexin A2–STAT3–Oncostatin M receptor axis drives phenotypic and mesenchymal changes in glioblastoma

**DOI:** 10.1186/s40478-020-00916-7

**Published:** 2020-04-05

**Authors:** Yuji Matsumoto, Tomotsugu Ichikawa, Kazuhiko Kurozumi, Yoshihiro Otani, Atsushi Fujimura, Kentaro Fujii, Yusuke Tomita, Yasuhiko Hattori, Atsuhito Uneda, Nobushige Tsuboi, Keisuke Kaneda, Keigo Makino, Isao Date

**Affiliations:** 1 Department of Neurological Surgery, Okayama University Graduate School of Medicine, Dentistry, and Pharmaceutical Sciences, Okayama, Japan; 2 Department of Neurosurgery, Kagawa Prefectural Central Hospital, Takamatsu, Kagawa Japan; 3 Department of Neurosurgery, McGovern Medical School, University of Texas Health Science Center at Houston, Houston, TX USA; 4 Department of Physiology, Okayama University Graduate School of Medicine, Dentistry and Pharmaceutical Sciences, Okayama, Japan

**Keywords:** ANXA2, OSMR, Invasion, Mesenchymal transition, Glioblastoma

## Abstract

Glioblastoma (GBM) is characterized by extensive tumor cell invasion, angiogenesis, and proliferation. We previously established subclones of GBM cells with distinct invasive phenotypes and identified annexin A2 (ANXA2) as an activator of angiogenesis and perivascular invasion. Here, we further explored the role of ANXA2 in regulating phenotypic transition in GBM. We identified oncostatin M receptor (OSMR) as a key ANXA2 target gene in GBM utilizing microarray analysis and hierarchical clustering analysis of the Ivy Glioblastoma Atlas Project and The Cancer Genome Atlas datasets. Overexpression of ANXA2 in GBM cells increased the expression of OSMR and phosphorylated signal transducer and activator of transcription 3 (STAT3) and enhanced cell invasion, angiogenesis, proliferation, and mesenchymal transition. Silencing of OSMR reversed the ANXA2-induced phenotype, and STAT3 knockdown reduced OSMR protein expression. Exposure of GBM cells to hypoxic conditions activated the ANXA2–STAT3–OSMR signaling axis. Mice bearing ANXA2-overexpressing GBM exhibited shorter survival times compared with control tumor-bearing mice, whereas OSMR knockdown increased the survival time and diminished ANXA2-mediated tumor invasion, angiogenesis, and growth. Further, we uncovered a significant relationship between ANXA2 and OSMR expression in clinical GBM specimens, and demonstrated their correlation with tumor histopathology and patient prognosis. Our results indicate that the ANXA2–STAT3–OSMR axis regulates malignant phenotypic changes and mesenchymal transition in GBM, suggesting that this axis is a promising therapeutic target to treat GBM aggressiveness.

## Introduction

Glioblastoma (GBM) is the most common and lethal primary brain malignancy in adults [23]. The defining aggressive hallmarks of GBM include abundant angiogenesis and marked proliferative and invasive behavior [12]. Genome-wide expression profiling has shown that GBM can be classified into classical, mesenchymal, neural, and proneural subtypes [36]. Among these, the mesenchymal subtype is characterized by a particularly aggressive phenotype with elevated invasive and angiogenic potential [5].

Although recent genetic analyses have shed light on the molecular alterations underlying GBM behaviors [4, 36], molecular targeted therapies have not yet led to improvements in the overall survival of GBM patients [10, 39]. One reason is the marked intratumoral genetic heterogeneity and plasticity exhibited by GBM [26], including induction of a mesenchymal transition after therapy with cytotoxic agents [3]. This tumor also frequently shows changes in biological features upon recurrence and progression. Therefore, elucidating the mechanisms underlying the phenotypic heterogeneity and transition is necessary to facilitate the development of curative therapies for GBM [12].

We have previously described two canine GBM cell lines, J3T-1 and J3T-2 [13, 20], that undergo annexin A2 (ANXA2)-regulated shifts in their angiogenesis and invasion phenotypes [12, 22, 24]. ANXA2 is a 36-kDa calcium-dependent phospholipid-binding protein [9] located mainly in the plasma membrane and cytoplasm, with low expression in the nucleus [7]. ANXA2 is upregulated in several tumors and plays critical roles in tumor development [6]. GBM cells often invade along anatomic brain structures, including blood vessels and white matter tracts [30]. We previously established two novel invasive GBM cell line models, J3T-1 and J3T-2 [13, 20], which imitated mimicked the angiogenic and invasive phenotypes of human GBM [22]. J3T-1 cells express high levels of ANXA2 and exhibit marked angiogenesis and invasion around the neovasculature, whereas J3T-2 cells express low ANXA2 levels and show a diffuse invasion pattern [13, 25]. We also showed that silencing of ANXA2 in J3T-1 cells (J3T-1shA) caused a switch to the diffuse invasion pattern, and conversely, overexpression of ANXA2 in J3T-2 cells (J3T-2A) induced a highly angiogenic phenotype [22]. Although these results indicated that ANXA2 could regulate the phenotypic shift of GBM, the molecular mechanisms by which this occurred remained unclear. In this study, we analyzed the gene expression profiles regulated by ANXA2 and its downstream pathways in GBM. We identified important roles for an ANXA2-induced signaling pathway involving signal transducer and activator of transcription 3 (STAT3) and oncostatin M receptor (OSMR) in regulating the phenotypic transition in GBM.

## Materials and methods

### Culture of cell lines and patient-derived GBM cells

The J3T-1 and J3T-2 cell lines were derived from the same parental canine GBM cells (J3T) and were characterized as previously reported [11, 13]. The J3T cell line was a gift from Dr. Michael E. Berens (Barrow Neurological Institute, Phoenix, AZ, USA) [2, 28]. J3T-1shA and J3T-2A were established as previously reported [22]. The following human GBM cell lines were provided as follows: A172 was from Dr. E. Antonio Chiocca (Brigham and Women’s Hospital, Boston, MA, USA); U87ΔEGFR and U251 were from Dr. Balveen Kaur (University of Texas Health Science Center, Houston, TX, USA); U87MG was purchased from the American Type Culture Collection (Manassas, VA, USA); LZN308 was gifted from Dr. Hioryuki Michiue (Okayama University, Okayama, Japan); and the patient-derived neurosphere GBM cells MGG8, MGG18, and MGG23 were a gift from by Dr. Hiroaki Wakimoto (Massachusetts General Hospital, Boston, MA, USA) and were cultured as previously described [37]. Human umbilical vein endothelial cells (HUVECs) were purchased from Takara Bio Inc. (Shiga, Japan) and cultured in EGM-2 BulletKit medium (Lonza, Basel, Switzerland). All other cell lines were cultured in Dulbecco’s Modified Eagle’s Medium with 10% fetal bovine serum, 100 U penicillin, and 0.1 mg/ml streptomycin at 37 °C in a 5% CO_2_ atmosphere. For hypoxic conditions, cells were cultured in a 1% O_2_ atmosphere or in medium containing 100 μM deferoxamine mesylate (Sigma-Aldrich, St. Louis, MO, USA). Cell lines were authenticated by Promega (Madison, WI, USA) using short tandem repeat profiling in December 2016.

### Human glioblastoma specimens

Forty GBM specimens suitable for qRT-PCR and immunohistochemical staining were obtained from 103 primary GBM patients treated at the Okayama University Hospital from 2006 to 2018. A summary of the characteristics of the forty primary GBM specimens is available in Additional file 1.

### Microarray assays

Total RNA was isolated from canine J3T-1, J3T-2, J3T-1shA, and J3T-2A cells using RNeasy kit (Qiagen, Santa Clarita, CA, USA) and samples were analyzed using a GeneChip Canine Genome 2.0 Array (Affymetrix, Santa Clara, CA, USA). The microarray analyses were performed by Takara Bio Inc. Briefly, biotinylated cRNA was synthesized from 250 ng total RNA using the GeneChip 3′ IVT PLUS Reagent Kit (Affymetrix), according to the manufacturer’s instructions. Biotinylated cRNA yields were checked with a NanoDrop ND-2000 spectrophotometer (Thermo Fisher Scientific, Scotts Valley, CA, USA). Following fragmentation, 15 μg of cRNA was hybridized for 16 h at 45 °C on a GeneChip Canine Genome 2.0 array, which were then washed and stained using the GeneChip Fluidics Station 450 instrument (Affymetrix). Arrays were scanned using the GeneChip Scanner 3000 7G (Affymetrix). Data from the single-array analyses were calculated using the Microarray Suite version 5.0 (MAS 5.0; Affymetrix) with the default settings and global scaling as the normalization methods. The trimmed mean target intensity of each array was arbitrarily set to 100. A significant change in gene expression was defined as an absolute fold change in expression of ≥2.0 with a *P* value of < 0.05 compared with appropriate controls. The microarray data were deposited in the Gene Expression Omnibus (GEO) under accession number GSE138374.

### Invasion assay

Two in vitro invasion assays were performed. Patient-derived GBM cells were seeded into 96-well Costar ultra-low attachment plates (Corning Inc., Corning, NY, USA) at 1.0 × 10^3^ cells/well in 25 μl of medium. The plates were briefly centrifuged to allow formation of central spheroids, and growth factor-reduced Matrigel was then added to each well (25 μg/insert; Becton Dickinson, Franklin Lakes, NJ, USA). Digital images of the spheroid midplanes were acquired with a BZ-8100 microscope (Keyence, Osaka, Japan). After incubation, the radius of invasion was defined as the distance farthest from the spheroid edge and was calculated using ImageJ software (http://rsb.info.nih.gov/ij/), as previously described [35, 44]. The second assay was performed using Corning BioCoat Matrigel® Invasion Chambers (24-well format; Corning Inc.) as previously described [25]. Each chamber was randomly counted at five high-power fields to determine the mean number of invaded cells.

### Animal experiments

Five- to six-week-old female BALB/c-nu/nu mice were purchased from CLEA Japan Inc. (Tokyo, Japan). GBM cells (2 × 10^5^) were stereotaxically injected into the right frontal lobe of anesthetized mice. Mice were euthanized when they exhibited neurological symptoms.

### Statistical analyses

All analyses were conducted using R version 3.5.2 (R Core Team (2018). R: A language and environment for statistical computing. R Foundation for Statistical Computing, Vienna, Austria. URL https://www.R-project.org/.) and GraphPad Prism 8 (GraphPad, San Diego, CA, USA). Graphed data are presented as the mean ± standard error (SEM). Differences between group means were evaluated using two-tailed Student’s t-tests or one-way ANOVA with Bonferroni’s post hoc test for multiple comparisons. Correlation analyses were assessed using Pearson’s correlation method. Survival curves were estimated using the Kaplan–Meier method and compared using a log-rank test. A *P* value of < 0.05 was considered statistically significant.

Additional details about the materials and methods are available in the supplementary materials and methods (Additional file 2: Supplementary materials and methods).

## Results

### Identification of ANXA2-regulated genes by microarray, in silico, and in vitro analyses

**Fig. 1:**
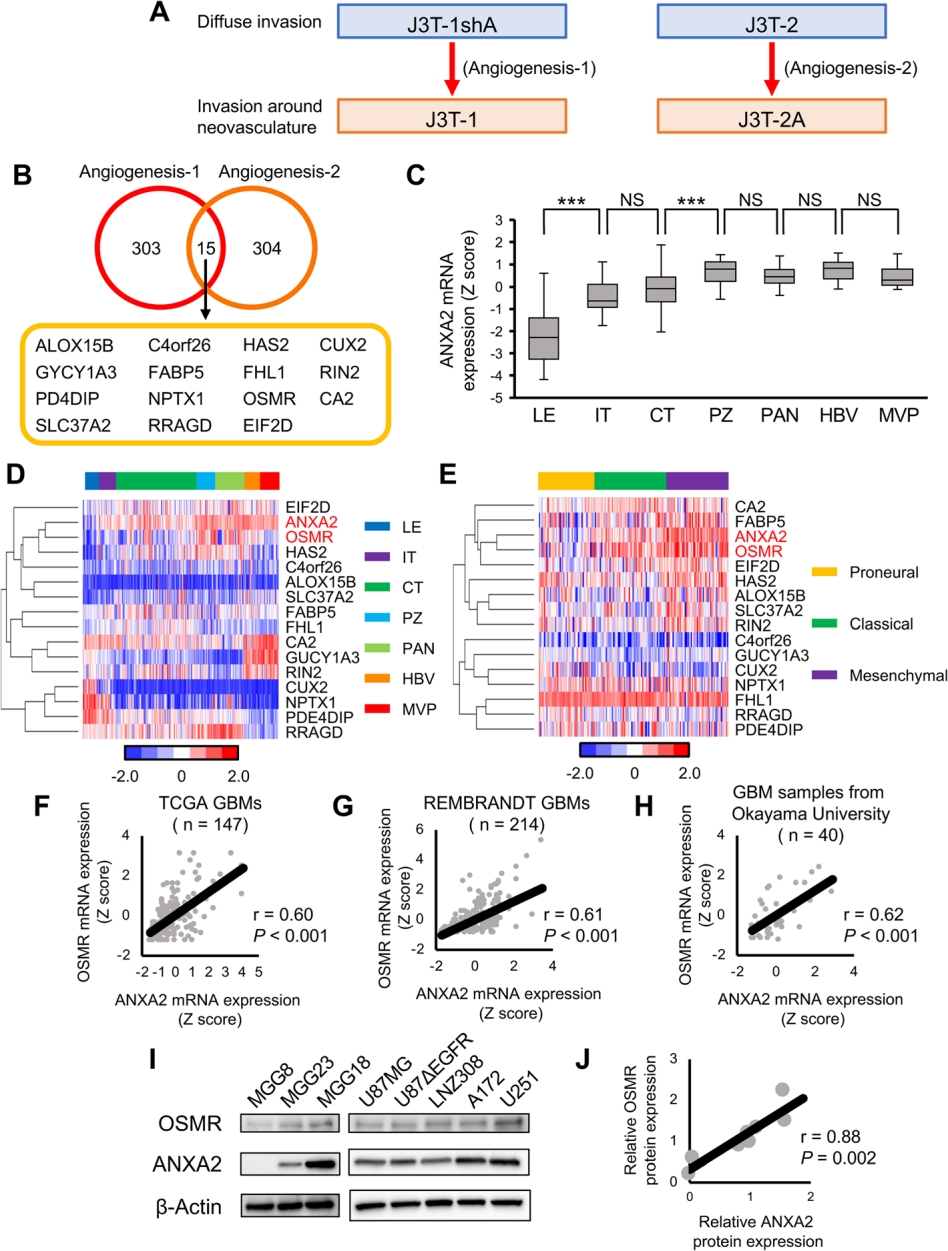
Identification of candidate ANXA2-regulated genes that promote angiogenesis and cell invasion. **a** Microarray analysis was performed to identify ANXA2-regulated genes that promote the angiogenesis–invasion phenotype. See text for the definitions of Angiogenesis-1 and Angiogenesis-2 genes. **b** Venn diagram of Angiogenesis-1, Angiogenesis-2, and shared genes identified from the analysis in (**a**). **c** Anatomical expression pattern of ANXA2 mRNA in the brain from the Ivy Glioblastoma Atlas Project dataset (*n* = 270). LE, leading edge; IT, infiltrating tumor; CT, cellular tumor; PZ, perinecrotic zone; PAN, pseudopalisading cells around necrosis; HBV, hyperplastic blood vessels; MVP, microvascular proliferation. Data are shown as the mean ± SEM. ***P < 0.001, NS not significant by one-way ANOVA with Bonferroni’s post hoc test. **d** Hierarchical clustering of genes related to angiogenesis and invasion ordered by anatomical location (Ivy GAP dataset, n = 270). **e** Hierarchical clustering of genes related to angiogenesis and invasion ordered by GBM molecular subtypes GBM (TCGA dataset, *n* = 143). **f–h** Pearson’s correlation tests of ANXA2 and OSMR mRNA expression in GBM datasets from TCGA (F, *n* = 147) and REMBRANDT (**g**, *n* = 214) and from GBM samples from Okayama University (**h**, *n* = 40). **i** Western blot analysis of ANXA2, OSMR, and β-actin in established human GBM cell lines and patient-derived GBM cells. **j** Pearson’s correlation test of ANXA2 and OSMR protein expression from the data shown in (**i**)

As noted above, J3T-1 and J3T-2A cells express elevated ANXA2 levels and exhibit a highly angiogenic and invasive phenotype, while J3T-2 and J3T-1shA cells express low levels of ANXA2 and exhibit a more diffuse invasive phenotype [22]. To identify genes regulated by ANXA2, we performed microarray analyses of these four cell lines. Genes regulated by ANXA2 were defined as those meeting both of the following two criteria: Angiogenesis-1 genes were expressed at > 2-fold higher levels in J3T-2A cells than in J3T-2 cells, and Angiogenesis-2 genes were expressed at > 2-fold in J3T-1 cells compared with J3T-1shA cells (Fig. 1a). The number of genes identified as satisfying the Angiogenesis-1 and Angiogenesis-2 criteria was 303 and 304, respectively, and 15 genes met both criteria (Fig. 1b).

The gene expression profile of GBM cells is known to depend on the anatomical location of the tumor. Thus, we examined the anatomical expression pattern of ANXA2 and these 15 ANXA2-regulated angiogenesis-invasion-related genes using the Ivy Glioblastoma Atlas Project (Ivy GAP) dataset. Consistent with our previous findings [22], high ANXA2 expression was observed in the perinecrotic zone, pseudopalisading cells around sites of necrosis, hyperplastic blood vessels, and microvascular proliferation (Fig. 1c). Clustering analysis of the genes using Ivy GAP dataset revealed that the oncostatin M receptor (OSMR) had the most similar anatomical expression pattern to ANXA2 (Fig. 1d).

Next, we validated the relationship between the expression of ANXA2 and the 15 identified genes in GBM molecular subtypes. Cluster analysis using The Cancer Genome Atlas (TCGA) dataset also revealed a close similarity between the expression patterns of ANXA2 and OSMR in the molecular subtypes (Fig. 1e). ANXA2 mRNA levels were significantly higher in samples of the mesenchymal subtype compared with the proneural and classical subtypes in the TCGA dataset (Additional file 3: Supplementary Fig. S1A). OSMR mRNA expression was also elevated in the mesenchymal subtype compared with the remaining subtypes, though the difference was not statistically significant between the classical subtype and the mesenchymal subtype (Additional file 3: Supplementary Fig. S1B). We further used bioinformatic analyses of single-cell RNA-Seq of primary human GBMs from the GSE57872 dataset [26].. Each GBM single-cell subtype was determined by a single sample gene set enrichment analysis [1, 38], and we found that ANXA2 expression was significantly elevated in the mesenchymal subtype compared with the remaining subtypes at the single-cell level (Additional file 3: Supplementary Fig. S1C). GSEA of the TCGA dataset confirmed significant enrichment of genes related to angiogenesis and cell invasion in mesenchymal subtypes (Additional file 4: Supplementary Fig. S2).

We next validated the relationship between the expression of ANXA2 and the 15 genes in human GBM specimens. Correlation analyses of the TCGA GBM dataset showed the strongest correlation was between ANXA2 and OSMR mRNA expression (Fig. 1f and Additional file 5: Supplementary Fig. S3), and this was confirmed by similar analyses of GBM datasets from the Repository for Molecular Brain Neoplasia Data (REMBRANDT) and Okayama University (Fig. 1g and h). Finally, we verified the correlation between ANXA2 and OSMR expression by performing western blot analysis of five human GBM cell lines (U87MG, U87ΔEGFR, LNZ308, A172, and U251) and three human GBM patient-derived cell lines (MGG8, MGG18 and MGG23). This analysis revealed a significant positive correlation between ANXA2 and OSMR protein levels (*r* = 0.88, *P* = 0.002; Fig. 1i and j).

Taken together, these data identify OSMR as a key ANXA2 target gene. The two genes have similar expression patterns with respect to anatomical location and molecular subtype, and exhibit significantly correlated expression at the mRNA and protein levels. Therefore, we examined whether ANXA2 regulates the phenotypic transition of GBM via OSMR.

### ANXA2 regulates mesenchymal transition, cell proliferation, and cell motility in GBM via OSMR

**Fig. 2:**
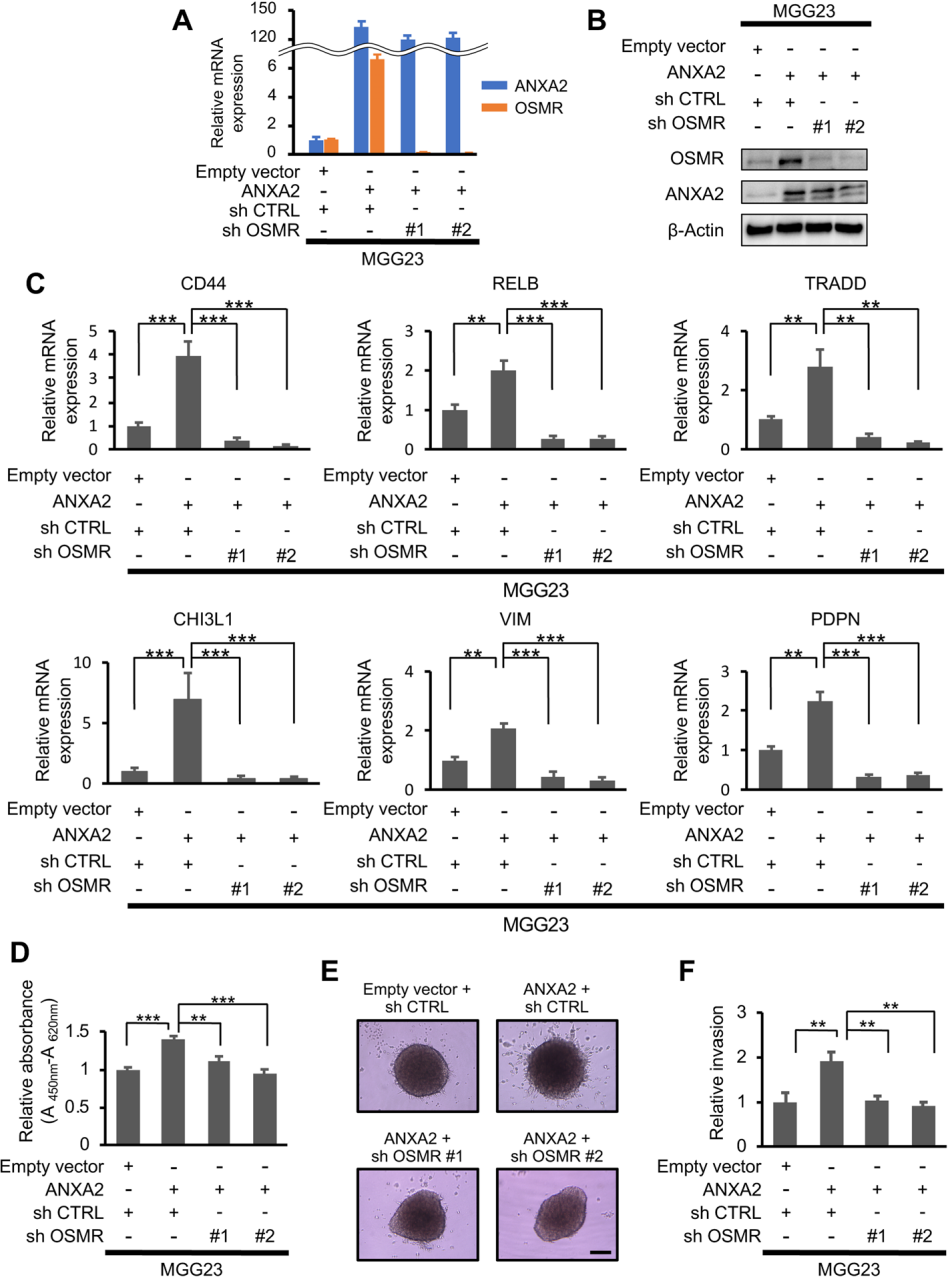
ANXA2- and OSMR-mediated regulation of cell proliferation, invasion, and mesenchymal transition in vitro. **a**, **b** qRT-PCR (**a**) and western blot analysis (**b**) of ANXA2 and OSMR expression in MGG23 cells with or without ANXA2 overexpression and concomitant OSMR knockdown. **c** qRT-PCR analysis of mesenchymal signature gene expression in MGG23 cells (*n* = 3). **d** WST-1 cell proliferation assay of MGG23 cells after 2 days incubation. Data were normalized to the control cells (*n* = 10). **e**, **f**| Matrigel invasion assay of MGG23 cells after 10 days. Data were normalized to the control cells (*n* = 4). Scale bar, 200 μm. Data are shown as the mean ± SEM. **P < 0.01, ***P < 0.001 by one-way ANOVA with Bonferroni’s post hoc test

GSEA of the GSE4412 dataset [8] revealed a positive association between high of OSMR or ANXA2 and expression of mesenchymal signature genes (Additional file 6: Supplementary Fig. S4A), supporting a role for OSMR in ANXA2-regulated functions. To investigate this further, we established stable MGG23 and U87MG GBM cell lines overexpressing ANXA2 in the presence or absence of concomitant shRNA-mediated OSMR silencing. Notably, ANXA2 overexpression alone in MGG23 cells strongly upregulated OSMR mRNA and protein expression (Fig. 2a and b) and increased transcription of mesenchymal genes (Fig. 2c. Moreover, silencing of OSMR virtually abolished the ANXA2-mediated increase in mesenchymal gene expression (Fig. 2c). Similar findings were observed in the U87MG cell lines (Additional file 6: Supplementary Fig. S4B–S4D). Silencing of OSMR alone in MGG23 cells decreased transcription of mesenchymal genes (Additional file 7: Supplementary Fig. S5A–S5C).

GSEA of the GSE4412 dataset revealed significant enrichment of genes related to cell proliferation and invasion in human GBM specimens with high expression of either ANXA2 or OSMR (Additional file 8: Supplementary Fig. S6A and S6B). Indeed, we found that while overexpression of ANXA2 in MGG23 cells significantly increased cell proliferation (Fig. 2d, *P* < 0.001), the increase was significantly suppressed by concomitant OSMR knockdown (Fig. 2d, *P* = 0.0016 and *P* < 0.001, respectively). Similar results were obtained in analyses of cell invasion using Matrigel invasion assays. Thus, invasion of MGG23 outside the core spheroid was significantly increased by ANXA2 overexpression (Fig. 2e and f, *P* = 0.006), but the increase was abolished by concomitant OSMR knockdown (Fig. 2e and f, *P* = 0.008 and 0.003, respectively). The same proliferation and invasion phenotypes were observed with U87MG cells (Additional file 9: Supplementary Fig. S7A**–**C). Silencing of OSMR alone in MGG23 cells reduced cell proliferation and invasion (Additional file 9: Supplementary Fig. S7D**–**F). Thus, the effects of ANXA2 on the proliferative and invasive phenotypes of GBM cells are mediated via OSMR.

### ANXA2 and OSMR modulate angiogenesis in vitro

**Fig. 3:**
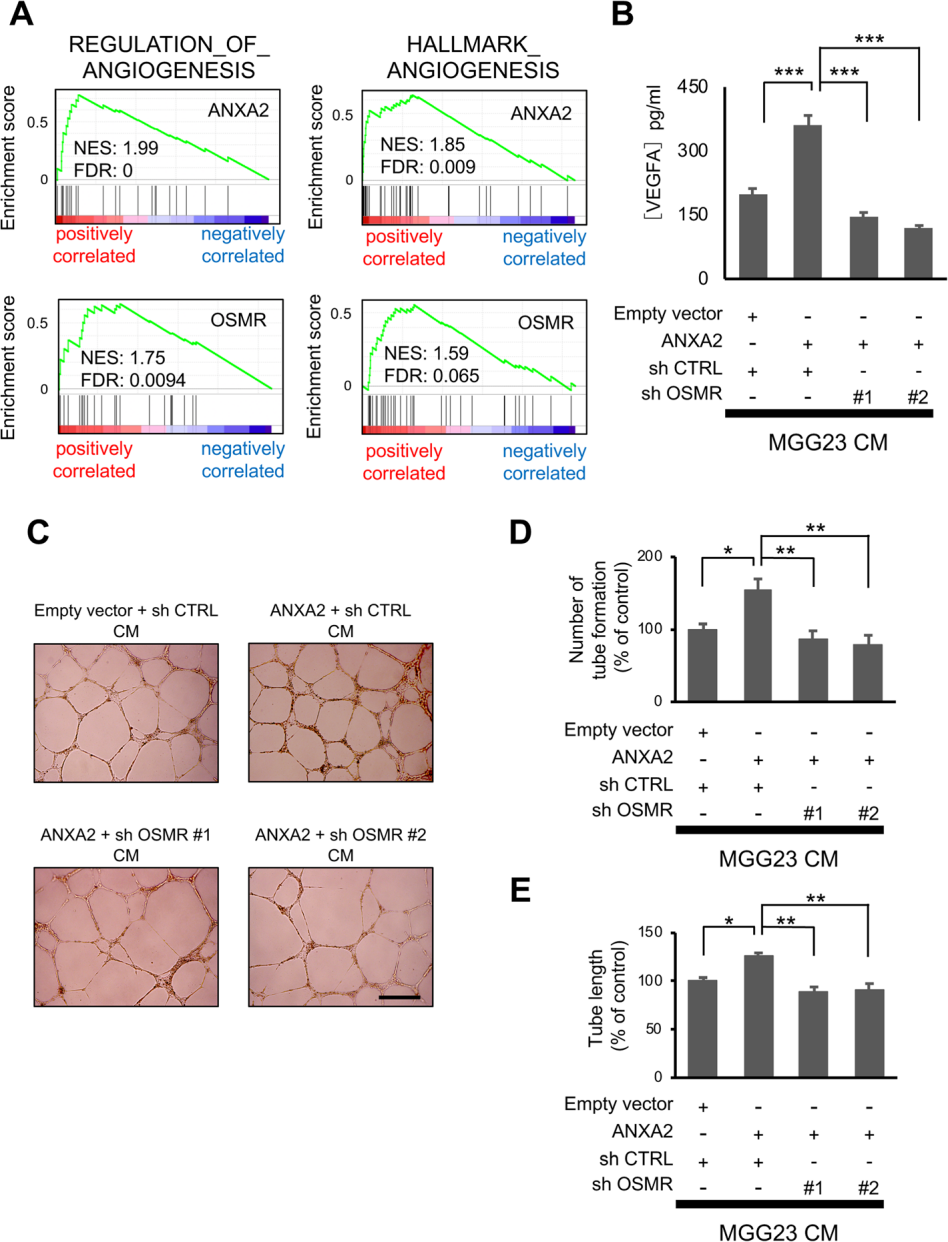
ANXA2- and OSMR-mediated regulation of angiogenesis in vitro. GSEA of angiogenesis-related genes and ANXA2 or OSMR expression in the GSE4412 dataset (*n* = 85). **b** ELISA analysis of VEGFA concentrations secreted by the indicated MGG23 cells incubation for 24 h in neural basal medium without growth factors (*n* = 8). **c–e** Tube formation assays of HUVECs after incubation for 20 h in growth factor-reduced Matrigel and conditioned media from the cells shown in (**b**). Representative images are shown in (**c**) and quantification of tube number and tubule length are shown in (**d**) and (**e**), respectively (*n* = 4). Scale bar, 500 μm. Data are shown as the mean ± SEM. *P < 0.05, **P < 0.01, ***P < 0.001 by one-way ANOVA with Bonferroni’s post hoc test

Our findings suggesting a role for OSMR in ANXA2-mediated regulation of angiogenesis were supported by GSEA analysis of dataset GSE4412, which revealed strong correlations between expression of angiogenesis-related genes and high ANXA2 or OSMR expression in human GBM (Fig. 3a). To investigate this directly, we first examined secretion of the angiogenic cytokine vascular endothelial cell growth factor A (VEGFA) by MGG23 cells incubated for 24 h in vitro. We found that VEGFA secretion was significantly elevated by ANXA2 overexpression but this was suppressed by simultaneous OSMR knockdown (Fig. 3b). As expected, similar results were obtained in HUVEC tube formation assays performed using conditioned medium from the same MGG23 cell lines. Thus, both the number of tubes formed and their length were increased by treatment with conditioned medium from ANXA2-overexpressing cells, while silencing of OSMR reversed these effects (Fig. 3d and e). These data indicate that ANXA2-mediated regulation of OSMR modulates angiogenesis in vitro*.*

### ANXA2 controls OSMR expression via phosphorylation of STAT3

**Fig. 4:**
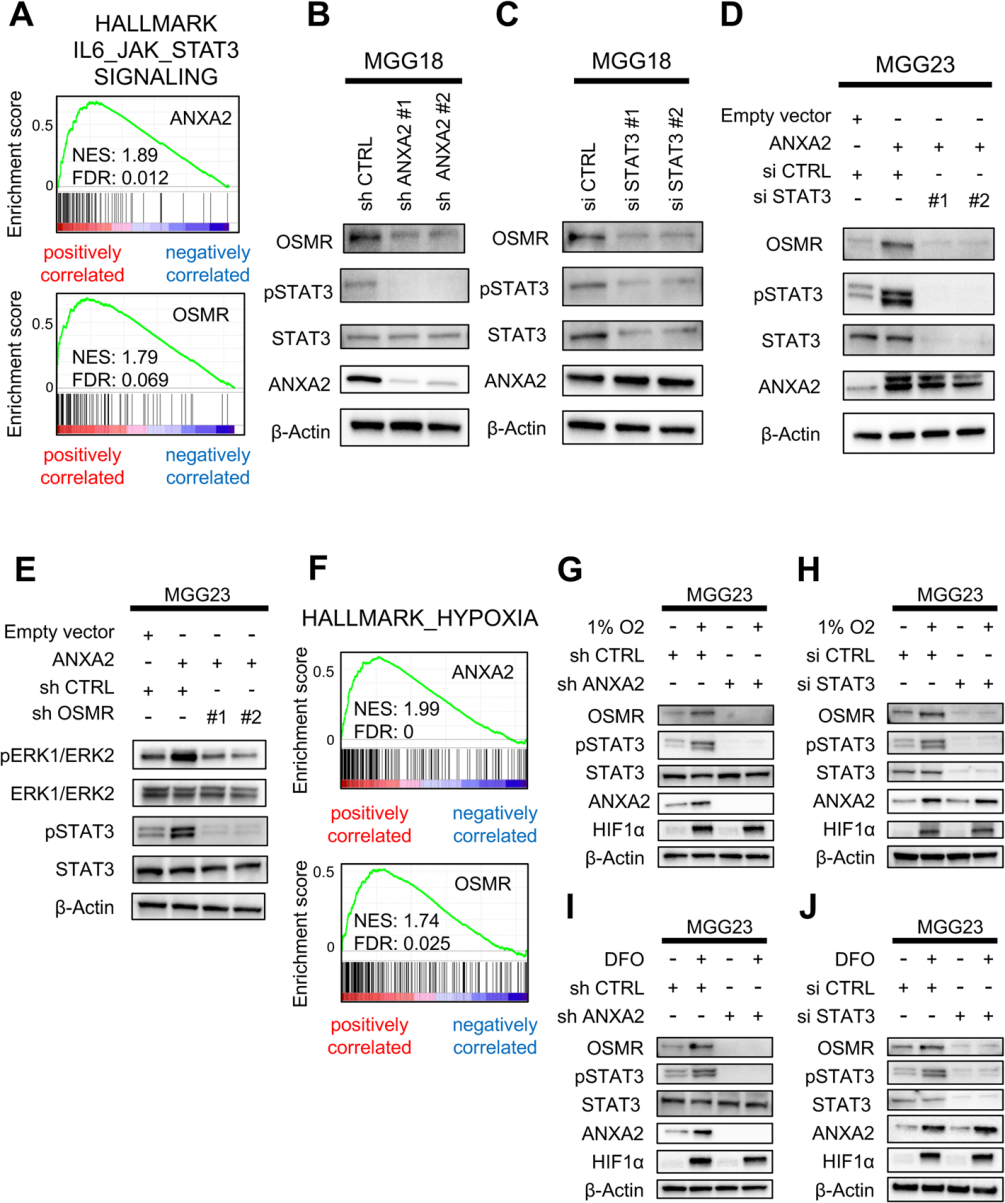
ANXA2–OSMR–STAT3 regulation and activation in GBM cells under normal and hypoxic conditions. **a** GSEA of IL6–JAK–STAT3 signaling genes and ANXA2 or OSMR expression in the GSE4412 dataset (*n* = 85). **b**, **c** Western blot analysis of the indicated proteins in MGG18 cells after ANXA2 (**b**) or STAT3 (**c**) knockdown. **d**, **e** Western blot analysis of the indicated proteins in MGG23 cells overexpressing ANXA2 with or without concomitant STAT3 (**d**) or OSMR (**e**) knockdown. **f** GSEA of hypoxia-associated genes and ANXA2 or OSMR expression in the GSE4412 dataset. **g–j** Western blot analysis of the indicated proteins in MGG23 cells overexpressing ANXA2 with or without concomitant OSMR knockdown. Cells were incubated in 1% O_2_ (**g, h**) or treated with deferoxamine mesylate (DFO) (**i, j**) for 24 h

Next, we sought to identify the mechanism by which ANXA2 induces OSMR expression in GBM cells. Previous studies have shown that ANXA2 increases the phosphorylation of STAT3 on tyrosine 705 (pSTAT3) [17], and that OSMR is a direct transcriptional target of STAT3 in GBM [14], suggesting that ANXA2 may upregulate OSMR expression by inducing STAT3 phosphorylation. In support of this, GSEA of GSE4412 showed a positive association between expression of interleukin (IL) –6–JAK–STAT3 pathway signature genes and high ANXA2 or OSMR expression in human glioma specimens (Fig. 4a). Western blot analysis of MGG18 cells, which express high endogenous levels of both ANXA2 and OSMR, revealed that depletion of ANXA2 suppressed both OSMR and pSTAT3 protein expression (Fig. 4b). STAT3 knockdown in MGG18 cells decreased the expression of OSMR but not ANXA2 (Fig. 4c). Moreover, STAT3 knockdown in MGG23 cells overexpressing ANXA2 also reduced OSMR protein expression (Fig. 4d). These findings suggest that ANXA2 regulates OSMR expression via phosphorylation of STAT3.

ANXA2 has been reported to affect breast cancer cell proliferation and invasion via activation of the ERK1/2 signaling pathway [43]. To investigate this, we performed western blot analysis of ERK1/2 and pERK1/2 in MGG23 cells and found that knockdown of either ANXA2 or OSMR reduced ERK1/2 phosphorylation (Additional file 10: Supplementary Fig. S8A and S8B). Interestingly, while overexpression of ANXA2 enhanced phosphorylation of both STAT3 and ERK, concomitant OSMR knockdown abolished the enhanced phosphorylation of both molecules (Fig. 4e). These results suggest that the ANXA2–STAT3–OSMR axis regulates the phenotypic transition of GBM cells via STAT3 and ERK signaling.

Hypoxia, common feature of the microenvironment of solid tumors, is a well-known inducer of angiogenesis, invasion, and mesenchymal transition in GBM [15, 32]. Therefore, we investigated the effects of hypoxia on activation of the ANXA2**–**STAT3–OSMR axis. GSEA of the GSE4412 demonstrated a positive association between expression of hypoxia pathway signature genes and high ANXA2 or OSMR expression in glioma specimens (Fig. 4f). Incubation of GBM cells in 1% O_2_ or treatment with deferoxamine mesylate, which mimics the effects of hypoxia by stabilizing expression a hypoxia-response transcription factor, increased the expression of ANXA2, OSMR, and pSTAT3 (Fig. 4g–j). Depletion of ANXA2 under the same conditions suppressed pSTAT3 and OSMR expression (Fig. 4g and i), while STAT3 silencing decreased expression of OSMR but not ANXA2 (Fig. 4h and j). These data suggest that hypoxia stimulates activation of the ANXA2–STAT3–OSMR axis.

### ANXA2 and OSMR induce a phenotypic transition of GBM cells in a mouse model

**Fig. 5:**
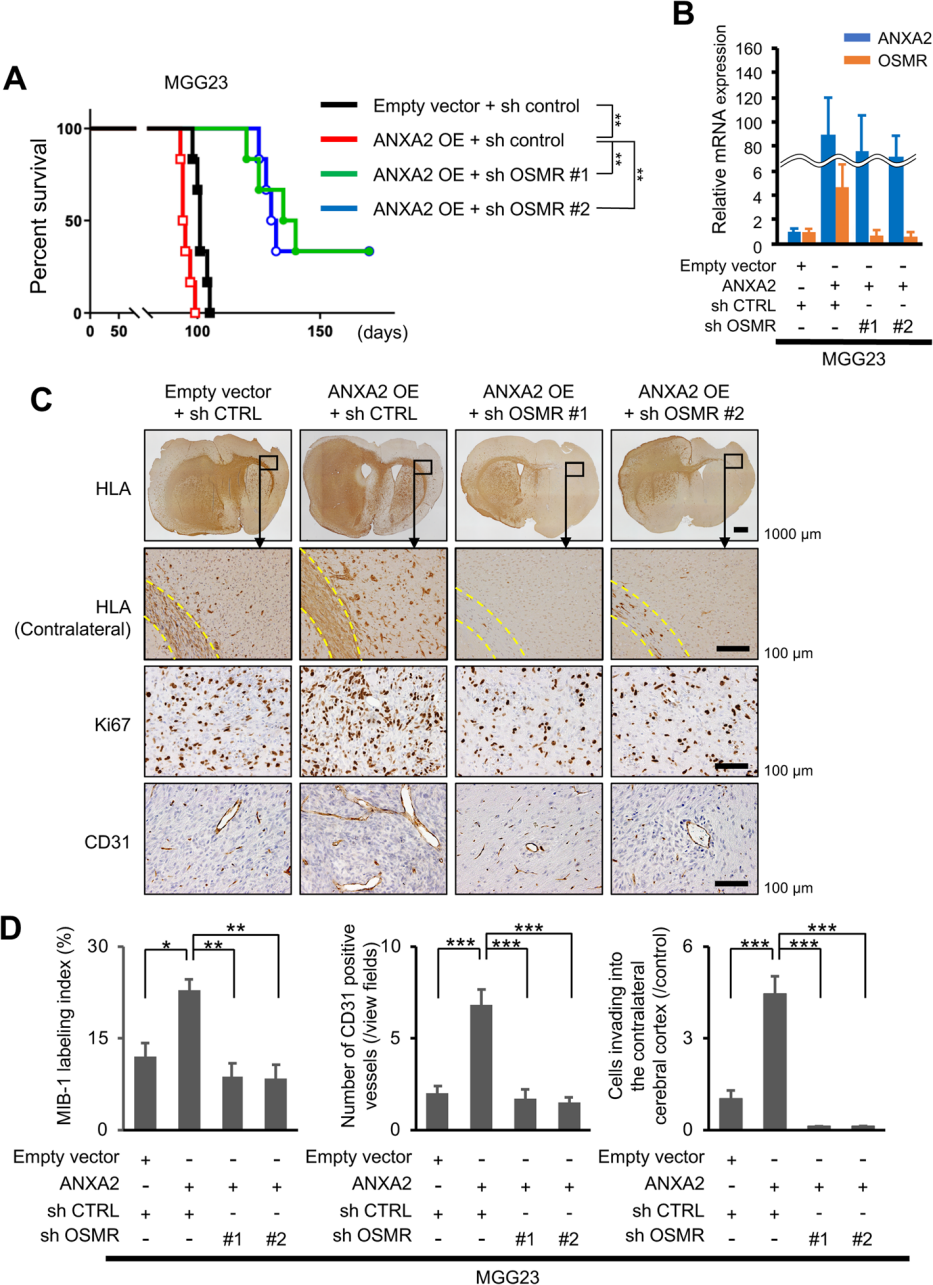
ANXA2- and OSMR-mediated regulation of tumor growth and phenotypic transition in a mouse xenograft model of GBM. **a** Kaplan–Meier survival curves of BALB/c-nu/nu mice injected intracranially with MGG23 cells expressing the indicated shRNAs (*n* = 6). **b** qRT-PCR using FFPE tissue sections of intracranial xenografts. **c**, **d** Immunohistochemical staining of human leukocyte antigen (HLA), Ki67, and CD31 in MGG23 tumors excised on day 91 after tumor cell injection (*n* = 5). Representative images (**c**) and quantification (**d**) of staining. Yellow dotted lines outline contralateral white matter tracts. Data are shown as the mean ± SEM. *P < 0.05, **P < 0.01, ***P < 0.001 by the log-rank test (**a**) or one-way ANOVA with Bonferroni’s post hoc test (**c**)

To determine whether our in vitro and in silico findings thus far translate to in vivo conditions, we examined the role of ANXA2 and OSMR in the growth of GBM xenografts in BALB/c-nu/nu mice. We injected one of four MGG23 cell lines into the brain of athymic mice: control cells, cells overexpressing ANXA2, or cells overexpressing ANXA2 and one of two OSMR-targeting shRNAs. While ANXA2 overexpression significantly shortened the survival of tumor-bearing mice compared with control mice (median survival = 94.5 days vs 101 days, *P* = 0.0094; Fig. 5a), concomitant silencing of OSMR significantly prolonged mouse survival (median survival = 131 or 137.5 days, both *P* = 0.0029; Fig. 5a). We confirmed the maintenance of ANXA2 overexpression and OSMR silencing in vivo after sacrifice by qRT-PCR using FFPE tissue sections of intracranial xenograft (Fig. 5b). Immunohistochemical examination of tumors excised on day 91 of the experiment showed spreading of control tumors from the injection site into the adjacent brain tissue and invasion along the corpus callosum (Fig. 5c). However, ANXA2-overexpressing tumors showed marked invasion into the cerebral cortex and increased cell proliferation (MIB-1 index) and angiogenesis (CD31 labeling; Fig. 5c and d). In keeping with the in vitro results, silencing of OSMR in ANXA2-overexpressing cells prevented the phenotypic shift (Fig. 5c and d).

To verify these findings, we injected mice intracranially with control U87ΔEGFR cells, which have high endogenous ANXA2 levels, or with U87ΔEGFR cells expressing ANXA2- or OSMR-targeting shRNAs. As expected, depletion of ANXA2 significantly increased survival (median survival: 13.5 vs. 17.5 or 17 days, *P* = 0.0058 or 0.014, respectively; Additional file 11: Supplementary Fig. S9A). Histological examination showed that control U87ΔEGFR tumors had well-defined borders with marked necrosis. In contrast, tumors formed by ANXA2-knockdown cells were smaller, exhibited no necrosis. and showed reduced cell proliferation and vessel formation (Additional file 11: Supplementary Fig. S9B and S9C). Depletion of OSMR had similar effects; namely, it significantly prolonged mouse survival (median survival: 13.5 vs. 21.5 or 22 days, both *P* = 0.0058; Additional file 11: Supplementary Fig. S9A), eliminated tumor necrosis, and diminished tumor growth, proliferation, and vessel density (Additional file 11: Supplementary Fig. S9B and S9C).

### ANXA2 and OSMR expression levels correlate with human glioma histopathology and patient prognosis

**Fig. 6:**
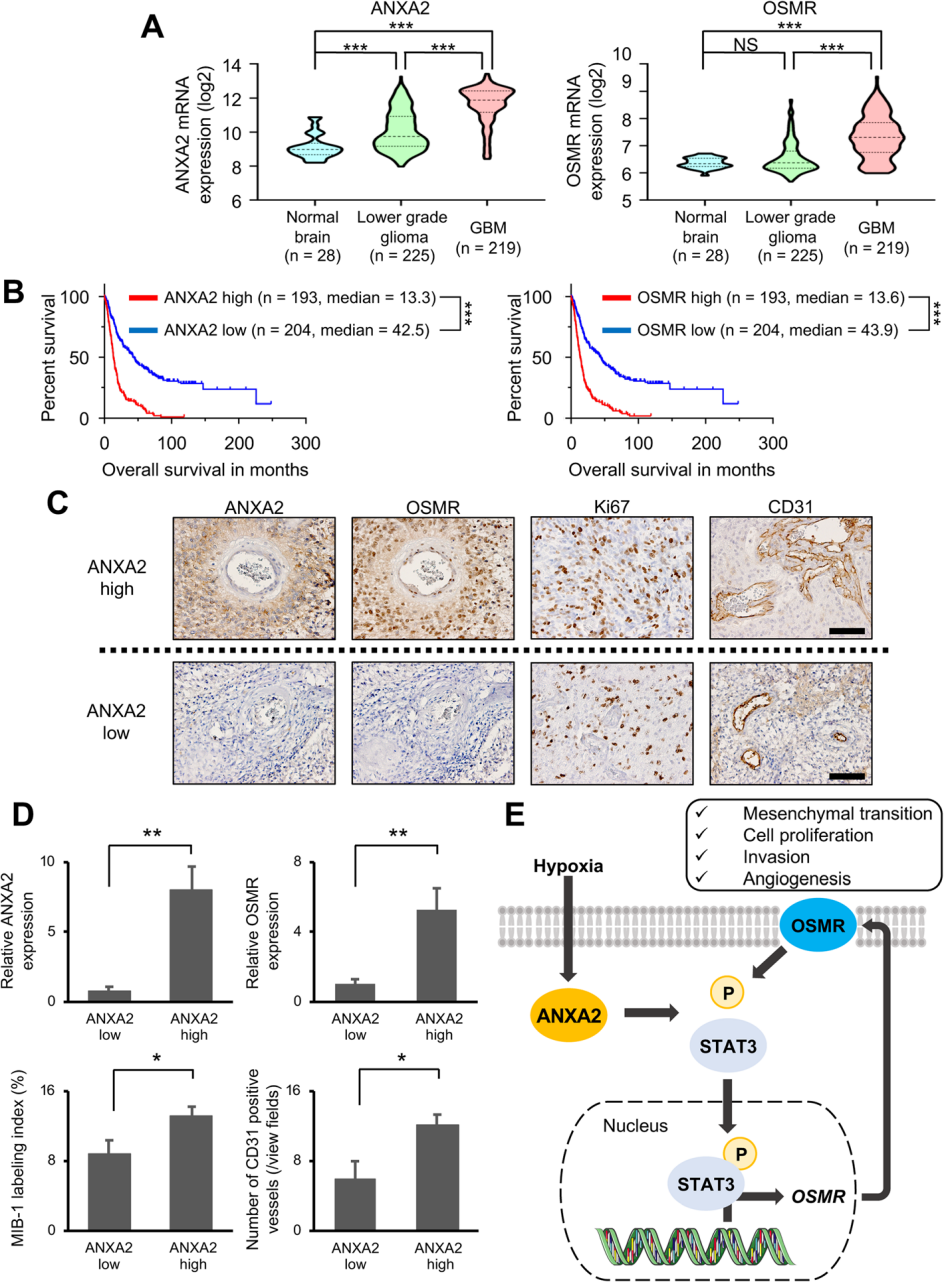
Correlations between ANXA2 and OSMR expression and human glioma histopathology and prognosis. **a** ANXA2 and OSMR mRNA expression in the REMBRANDT dataset (normal brain *n* = 28, lower grade glioma *n* = 225, GBM *n* = 219). **b** Kaplan–Meier overall survival curves of patients in the REMBRANDT glioma dataset stratified by high or low ANXA2 and OSMR mRNA levels (*n* = 397). **c, d** Analysis of patient tumor samples selected based on high or low ANXA2 mRNA levels. **c** Representative images of OSMR, CD31, and Ki67 staining and (**d**) quantification of ANXA2 and OSMR expression, cell proliferation (MIB-1 index), and angiogenesis (CD31 expression) (*n* = 10). Scale bar, 100 μm. **e** Schematic showing proposed model of phenotypic transition regulated by the ANXA2–STAT3–OSMR axis in GBM. Hypoxic conditions increase ANXA2 expression, STAT3 phosphorylation and nuclear localization, and OSMR expression, resulting in mesenchymal phenotypic changes such as prominent cell proliferation, invasion, and angiogenesis. Data are shown as the mean ± SEM. *P < 0.05, **P < 0.01, ***P < 0.001, NS not significant by one-way ANOVA with Bonferroni’s post hoc test (**a**), log-rank test (**b**), or two-tailed Student’s t-test (**d**)

Finally, we assessed the clinical significance of ANXA2 and OSMR expression on tumor growth and patient prognosis by analyzing the REMBRANDT, TCGA, and Chinese Glioma Genome Atlas (CGGA) dataset. In the REMBRANDT dataset, we found that ANXA2 and OSMR expression were associated with the histopathologic grade of glioma, and higher expression was observed in GBM than in lower grade glioma (Fig. 6a). In addition, high expression (> median level) of ANXA2 or OSMR was significantly associated with poor survival in the glioma patients (Fig. 6b). Focusing on the GBM dataset, ANXA2 expression was significantly associated with poorer survival in the CGGA GBM dataset (ANXA2 high, median survival = 14.4 months; ANXA2 low, median survival = 19.8 months; *P* = 0.01), though the difference was not statistically significant in the TCGA GBM dataset (ANXA2 high, median survival = 12.6 months; ANXA2 low, median survival = 14.9 months; *P* = 0.097) (Additional file 12: Supplementary Fig. S10A). In addition, OSMR expression was significantly associated with poor survival in the TCGA GBM dataset (OSMR high, median survival = 12.6 months; OSMR low, median survival = 14.5 months; *P* = 0.0097) and the CGGA GBM dataset (OSMR high, median survival = 14.2 months; OSMR low, median survival = 19.2; *P* = 0.017) (Additional file 12: Supplementary Fig. S10B). Next, we screened a series of human GBM specimens obtained from Okayama University (*n* = 40) and selected 10 samples each with the highest and lowest ANXA2 mRNA expression. Immunohistochemical examination of these 20 samples showed that tumors with high ANXA2 mRNA levels also showed higher ANXA2 and OSMR protein expression (*P* = 0.0015 and *P* = 0.0041, respectively), had a higher MIB-1 labeling index (*P* = 0.031), and exhibited more extensive vascularity (*P* = 0.021) compared with samples expressing low ANXA2 levels (Fig. 6c and d). These results suggest that ANXA2 and OSMR play an important role in the malignant histopathological phenotype of GBM and patient prognosis.

## Discussion

In this study, we sought to identify ANXA2-regulated genes in GBM. Microarray analysis identified 15 genes related to the angiogenesis–invasion phenotype. Analysis of Ivy GAP and TCGA datasets revealed strong correlations between OSMR and ANXA2 expression, and the functional roles of ANXA2 and OSMR in cell proliferation, invasion, and angiogenesis were confirmed in vitro and in vivo. Collectively, our data suggest that ANXA2 regulates OSMR expression via STAT3 phosphorylation, which drives the transition to a mesenchymal phenotype with prominent cell proliferation, invasion, and angiogenesis (Fig. 6e).

Although some recent studies have reported that survival time of GBM patients is associated with the extent of tumor resection [40], aggressive invasion and infiltration is a characteristic of GBM that makes it difficult to resect completely. The mesenchymal GBM subtype is particularly aggressive, with elevated invasive and angiogenic potential [5]. Recently, Puchalski et al. examined the transcriptional profiles of GBM subtype according to the anatomical regions; they identified leading edges as neural subtype, infiltrative tumors as neural/proneural, central tumor regions as either classical or neural/proneural subtypes, and pseudopalisading cells around necrotic areas and microvascular proliferation as almost exclusively mesenchymal subtype [27]. Although some factors that regulate anatomically related gene expression in GBM have been reported, the detailed mechanisms remain unclear [27, 34].

Our previous work suggested that ANXA2 may be involved in the shift of GBM towards an invasive phenotype [22]. Kling et al. reported that ANXA2 is at the apex of a regulatory cascade that determines GBM mesenchymal transition, and that ANXA2 knockdown led to a reduction in phosphorylated STAT3 and suppression of mesenchymal gene expression, cell proliferation, and invasion [17]. In the present study, we further reveal the mechanism of ANXA2 regulation of these events via OSMR.

OSMR is a member of the type I cytokine receptor family and forms a heterodimer with the common signal transducer gp130, which is shared with other members of the IL-6 family, including leukemia inhibitory factor (LIF), IL-6, IL-11, ciliary neurotrophic factor, and cardiotrophin-1 [21]. Previous studies have evaluated the role of OSMR and its ligand OSM in a range of cancers, including glioma. The OSM–OSMR axis is known to drive the epithelial–mesenchymal transition and is associated with poor survival [19, 31, 33, 41]. In squamous cell carcinoma, OSMR overexpression activates cell-autonomous feed-forward signaling that induces further expression of OSMR and OSM, leading to a pro-malignant phenotype [18]. Repovic et al. reported that OSM induction of VEGF expression is a unique property of OSM that is not shared with other IL-6 family members [29]. Jahani-Asl et al. showed that OSMR is a required co-receptor for EGFR variant III, which allows constitutive activation and plays a prominent role in GBM tumorigenesis [14]. In the present study, we found that ANXA2 overexpression activates STAT3, OSMR, and ERK, resulting in enhanced cell proliferation, invasion, angiogenesis, and mesenchymal transition.

Hypoxia is a well-recognized component of the tumor microenvironment and a known pathogenic driver in a variety of tumors, including GBM [42]. Hypoxia induces activation of the STAT3 pathway [16], which leads to enhanced expression of angiogenesis and invasion genes. However, little is known about the function of ANXA2 and OSMR in GBM under hypoxic conditions. We showed here that hypoxia induces ANXA2 and OSMR expression, thereby providing novel insight into the malignant phenotypic change in GBM. GSK2330811, a humanized anti-OSM blocking antibody, was recently tested in clinical trials for the treatment of systemic sclerosis (NCT03041025) and in a preclinical study of tumor-bearing mice [18]. We speculate that GSK2330811 may have utility in suppressing ANXA2–STAT3–OSMR signaling, thereby reducing malignant progression and improving patient survival.

In conclusion, our findings suggest that the ANXA2–STAT3–OSMR axis plays a crucial role in the regulation of the molecular phenotype and pathogenesis of GBM. The axis regulates the aggressiveness of GBM, including invasion, proliferation, angiogenesis, and mesenchymal transition, suggesting that inhibition of this axis may be an attractive therapeutic strategy for this tumor.

## Supplementary information

**Additional file 1.** List of forty primary GBM cases.

**Additional file 2.** Supplementary materials and methods.

**Additional file 3: Supplementary Figure S1**. ANXA2 and OSMR mRNA expression in the three GBM subtypes.

**Additional file 4: Supplementary Figure S2**. GSEA enrichment plots of angiogenesis and invasion signatures in the four GBM subtypes.

**Additional file 5: Supplementary Figure S3**. Correlations between mRNA expression of ANXA2 and 15 genes associated with the high angiogenesis–invasion phenotype (OSMR is not shown).

**Additional file 6: Supplementary Figure S4**. ANXA2 and OSMR modulate the mesenchymal transition of GBM cells in vitro.

**Additional file 7: Supplementary Figure S5**. OSMR alone modulates the mesenchymal transition of GBM cells in vitro.

**Additional file 8: Supplementary Figure S6**. GSEA enrichment plots of GBM patients expressing high versus low ANXA2 and OSMR mRNA levels.

**Additional file 9: Supplementary Figure S7**. Control of GBM cell proliferation and invasion by ANXA2 and OSMR.

**Additional file 10: Supplementary Figure S8**. Effect of ANXA2 and OSMR knockdown on STAT3 and ERK signaling in U87ΔEGFR cells.

**Additional file 11: Supplementary Figure S9**. Effect of ANXA2 and OSMR knockdown on GBM xenograft growth and phenotypic transition in mice.

**Additional file 12: Supplementary Figure S10**. Kaplan–Meier overall survival curves of patients in the TCGA GBM dataset and the CGGA GBM dataset stratified by high or low ANXA2 and OSMR mRNA levels.

**Additional file 13.** Supplementary figure legends.

## Data Availability

The accession number for the gene expression data reported in this paper is GSE138374.
