# Supplementary material for: Annexin A2–STAT3–Oncostatin M receptor axis drives phenotypic and mesenchymal changes in glioblastoma
Source: Acta Neuropathol Commun. 2020 Apr 5;8:42. doi: 10.1186/s40478-020-00916-7 (PMC7132881; doi:10.1186/s40478-020-00916-7)
Supplement: Supplementary file 2 — Additional file 2. Supplementary materials and methods. [file 40478_2020_916_MOESM2_ESM.docx]

**Supplementary materials and methods**

***Computational analysis***

For analysis of human GBM data, we downloaded gene expression and survival datasets from The Cancer Genome Atlas (TCGA) *via* the Project Betastatis webpage ([www.betastasis.com](http://www.betastasis.com)), GlioVis (http://gliovis.bioinfo.cnio.es/), and cBioPortal [1, 3]. A dataset from the Repository of Molecular Brain Neoplasia Database (REMBRANDT) and the Chinese Glioma Genome Atlas (CGGA) was downloaded from GlioVis. Dataset GSE4412 [2] was downloaded from the GEO database and analyzed using Gene Set Enrichment Analysis (<http://www.broadinstitute.org/gsea/index.jsp>). The normalized enrichment score and false discovery rate were used to determine statistical significance, as previously described [11]. For cluster analysis, datasets from the Ivy Glioblastoma Atlas Project (<http://glioblastoma.alleninstitute.org/>) and TCGA were used. Clustering was performed using CLUSTER 3.0 software (<http://bonsai.hgc.jp/~mdehoon/software/cluster/software.htm>) and Java TreeView (<http://jtreeview.sourceforge.net/>).

***Plasmids, RNA interference, and lentiviruses***

The human ANXA2 plasmids were purchased from GeneCopoeia (Rockville, MD, USA) and the open reading frame was cloned into pTomo vector, a gift from Dr. Inder Verma [5] (# 26291; Addgene, Cambridge, MA, USA). Human STAT3-targeting and negative control siRNAs were obtained from Thermo Fisher Scientific and transfected into cells using Lipofectamine® RNAiMAX Transfection Reagent (Thermo Fisher Scientific). Lentiviruses encoding human OSMR-targeting and scrambled shRNAs were prepared using plasmids pLKO.1 (a gift from Dr. David Root [6]; # 10879, Addgene), psPAX2, and pMD2.G (# 12260 and # 12259, Addgene, respectively; both gifts from Dr. Didier Trono), 293FT cells, and *Trans*IT®-LT1 Reagent (Mirus, Madison, WI, USA), according to manufacturer’s recommendations.

***RNA isolation, cDNA synthesis, and qRT-PCR***

Total RNA was isolated from cell lines, FFPE tissue sections of the intracranial xenograft, or human glioblastoma specimens, and cDNA was synthesized with a High-Capacity cDNA Reverse Transcription Kit (Invitrogen, Carlsbad, CA, USA). qPCR was performed using the StepOnePlus^TM^ System (Life Technologies, Carlsbad, CA, USA) with β-actin mRNA as an internal control. Primers specific for β-actin, ANXA2, and OSMR were designed using the Primer3 Plus software (http://www.bioinformatics.nl/cgibin/primer3plus/primer3plus.cgi) and synthesized by Integrated DNA Technologies (Coralville, IA, USA). Primers specific for mesenchymal signature genes were constructed as previously reported [7]. The primer sequences are as follows:

β-actin: forward, 5′-GGACTTCGAGCAAGAGATGG-3′;

reverse, 5′-AGCACTGTGTTGGCGTACAG-3′.

ANXA2: forward, 5′-GCCATCAAGACCAAAGGTGT-3′;

reverse, 5′-AAAATCACCGTCTCCAGGTG-3′.

OSMR: forward, 5′-GGAATGTGCCACACACTTTG-3′;

reverse, 5′-ACATTGGTGCCTTCTTCCAC-3′.

CD44: forward, 5′-CTGATCATCTTGGCATCCCT-3′;

reverse, 5′-AGCTTTTTCTTCTGCCCACA-3′.

RELB: forward, 5′-CAGCCTCGTGGGGAAAGAC-3′;

reverse, 5′-GCCCAGGTTGTTAAAACTGTGC-3′.

TRADD: forward, 5′-GCTGTTTGAGTTGCATCCTAGC-3′;

reverse, 5′-CCGCACTTCAGATTTCGCA-3′.

CHI3L1: forward, 5′-CTCAAGAACAGGAACCCCAA-3′;

reverse, 5′-TCTGGGTGTTGGAGGCTATC-3′.

VIM: forward, 5′-TGCCCTTAAAGGAACCAATG-3′;

reverse, 5′-GCTTCAACGGCAAAGTTCTC-3′.

PDPN: forward, 5′-ACCAGTCACTCCACGGAGAAA-3′;

reverse, 5′-GGTCACTGTTGACAAACCATCT-3′.

***Western blot analysis***

Cell lysis, SDS-PAGE, protein transfer and western blot analyses were carried out as previously described [8]. In brief, membranes were blocked in 5% skim milk and incubated overnight at 4°C with the following primary antibodies: anti-β-actin (Sigma-Aldrich, St. Louis, MO, USA; A5441, 1:5000), anti-ANXA2 (Invitrogen, #03-4400, 1:1000), anti-OSMR (Bio-Rad, Hercules, CA, USA; VPA00324, 1:1000), anti-STAT3 (Cell Signaling Technology, Danvers, MA, USA; #9139, 1:1000), anti-phospho-STAT3 (Cell Signaling Technology, #4093, 1:2000), anti-HIF-1α (Cell Signaling Technology, #3716, 1:1000), anti-ERK1/2 (Cell Signaling Technology, #9102, 1:1000), and anti-phospho-ERK1/2 (Cell Signaling Technology, #9101, 1:1000). The secondary antibodies were horseradish peroxidase (HRP)-conjugated anti-mouse IgG (Cell Signaling Technology, #7076, 1:5000) and HRP-conjugated anti-rabbit IgG (Cell Signaling Technology, #7074, 1:5000). HRP signals were visualized using the ECL^®^ Prime Western Blotting Detection or ECL^®^ Select Western Blotting Detection systems (GE Healthcare, Little Chalfont, UK) and analyzed using the VersaDoc^®^ molecular imaging system (Bio-Rad).

***Tube formation assay***

Growth factor-reduced Matrigel (50 μl) was thawed on ice, added to wells of a 96-well plate, and incubated at 37°C for 30 min to allow polymerization. HUVECs were seeded at 2.0 × 10^4^ cells per well in 100 μl of conditioned medium from GBM cells cultured at 37°C for 24 h. Plates containing HUVECs were then incubated at 37°C for 20 h, observed with a BZ-8100 microscope (Keyence, Osaka, Japan), and evaluated using the Angiogenesis Analyzer plugin (<http://image.bio.methods.free.fr/ImageJ/?Angiogenesis-Analyzer-for-ImageJ&lang=en>) for ImageJ [10].

***Immunohistochemistry***

Immunohistochemistry was carried out as previously described [8] using the following primary antibodies: anti-ANXA2 (Invitrogen, #03-4400, 1:100), anti-OSMR (LifeSpan BioSciences, Seattle, WA, USA; LS-B11477, 1:100), anti-HLA (Abcam, Cambridge, UK; ab70328, 1:100), anti-CD31 (Abcam, ab28364, 1:50); and anti-Ki67 (Leica Biosystems, Wetzlar, Germany; NCL-L-Ki67-MM1, 1:150). Antibody binding was revealed using a Dako Envision^®^+ System-HRP Kit in accordance with the manufacturer’s protocol (DakoCytomation, Carpentaria, CA, USA) and the sections were counterstained with hematoxylin. Samples were observed with a BZ-8100 microscope.

ANXA2 and OSMR immunoreactivity was expressed as the percentage of the total tissue area with positive staining and was measured using the Colour Deconvolution plugin [9] for ImageJ [4]. The MIB-1 labeling index was also evaluated using the same plugin. The three most vascularized areas in the tumor were visualized at low magnification (×40), and in each view-field, CD31-positive vessels were counted in a representative area under high-magnification (×200)[12].

***Proliferation assay***

Glioma cell proliferation was measured using a water-soluble tetrazolium-1 (WST-1) assay according to the manufacturer’s protocol (Roche, Mannheim, Germany).

***ELISA***

Human VEGFA in supernatant samples was quantified using a specific Quantikine ELISA Kit (R&D Systems, Minneapolis, MN, USA) according to the manufacturer’s instructions. Samples of conditioned medium were collected from patient-derived GBM cell cultures incubated in 60-mm plates in neural basal medium without growth factors for 24 h.

**References**

1 Cerami E, Gao J, Dogrusoz U, Gross BE, Sumer SO, Aksoy BA, Jacobsen A, Byrne CJ, Heuer ML, Larsson Eet al (2012) The cBio cancer genomics portal: an open platform for exploring multidimensional cancer genomics data. Cancer discovery 2: 401-404 Doi 10.1158/2159-8290.Cd-12-0095

2 Freije WA, Castro-Vargas FE, Fang Z, Horvath S, Cloughesy T, Liau LM, Mischel PS, Nelson SF (2004) Gene expression profiling of gliomas strongly predicts survival. Cancer Res 64: 6503-6510 Doi 10.1158/0008-5472.Can-04-0452

3 Gao J, Aksoy BA, Dogrusoz U, Dresdner G, Gross B, Sumer SO, Sun Y, Jacobsen A, Sinha R, Larsson Eet al (2013) Integrative analysis of complex cancer genomics and clinical profiles using the cBioPortal. Science signaling 6: pl1 Doi 10.1126/scisignal.2004088

4 Ishida J, Kurozumi K, Ichikawa T, Otani Y, Onishi M, Fujii K, Shimazu Y, Oka T, Shimizu T, Date I (2015) Evaluation of extracellular matrix protein CCN1 as a prognostic factor for glioblastoma. Brain Tumor Pathol 32: 245-252 Doi 10.1007/s10014-015-0227-3

5 Marumoto T, Tashiro A, Friedmann-Morvinski D, Scadeng M, Soda Y, Gage FH, Verma IM (2009) Development of a novel mouse glioma model using lentiviral vectors. Nat Med 15: 110-116 Doi 10.1038/nm.1863

6 Moffat J, Grueneberg DA, Yang X, Kim SY, Kloepfer AM, Hinkle G, Piqani B, Eisenhaure TM, Luo B, Grenier JKet al (2006) A lentiviral RNAi library for human and mouse genes applied to an arrayed viral high-content screen. Cell 124: 1283-1298 Doi 10.1016/j.cell.2006.01.040

7 Murata H, Yoshimoto K, Hatae R, Akagi Y, Mizoguchi M, Hata N, Kuga D, Nakamizo A, Amano T, Sayama Tet al (2015) Detection of proneural/mesenchymal marker expression in glioblastoma: temporospatial dynamics and association with chromatin-modifying gene expression. J Neurooncol 125: 33-41 Doi 10.1007/s11060-015-1886-y

8 Otani Y, Ichikawa T, Kurozumi K, Inoue S, Ishida J, Oka T, Shimizu T, Tomita Y, Hattori Y, Uneda Aet al (2018) Fibroblast growth factor 13 regulates glioma cell invasion and is important for bevacizumab-induced glioma invasion. Oncogene 37: 777-786 Doi 10.1038/onc.2017.373

9 Ruifrok AC, Johnston DA (2001) Quantification of histochemical staining by color deconvolution. Anal Quant Cytol Histol 23: 291-299

10 Schneider CA, Rasband WS, Eliceiri KW (2012) NIH Image to ImageJ: 25 years of image analysis. Nat Methods 9: 671-675

11 Subramanian A, Tamayo P, Mootha VK, Mukherjee S, Ebert BL, Gillette MA, Paulovich A, Pomeroy SL, Golub TR, Lander ESet al (2005) Gene set enrichment analysis: a knowledge-based approach for interpreting genome-wide expression profiles. Proc Natl Acad Sci U S A 102: 15545-15550 Doi 10.1073/pnas.0506580102

12 Sun H, Guo D, Su Y, Yu D, Wang Q, Wang T, Zhou Q, Ran X, Zou Z (2014) Hyperplasia of pericytes is one of the main characteristics of microvascular architecture in malignant glioma. PLoS One 9: e114246 Doi 10.1371/journal.pone.0114246
