# Supplementary figures and images for: Annexin A2–STAT3–Oncostatin M receptor axis drives phenotypic and mesenchymal changes in glioblastoma
Source: Acta Neuropathol Commun. 2020 Apr 5;8:42. doi: 10.1186/s40478-020-00916-7 (PMC7132881; doi:10.1186/s40478-020-00916-7)

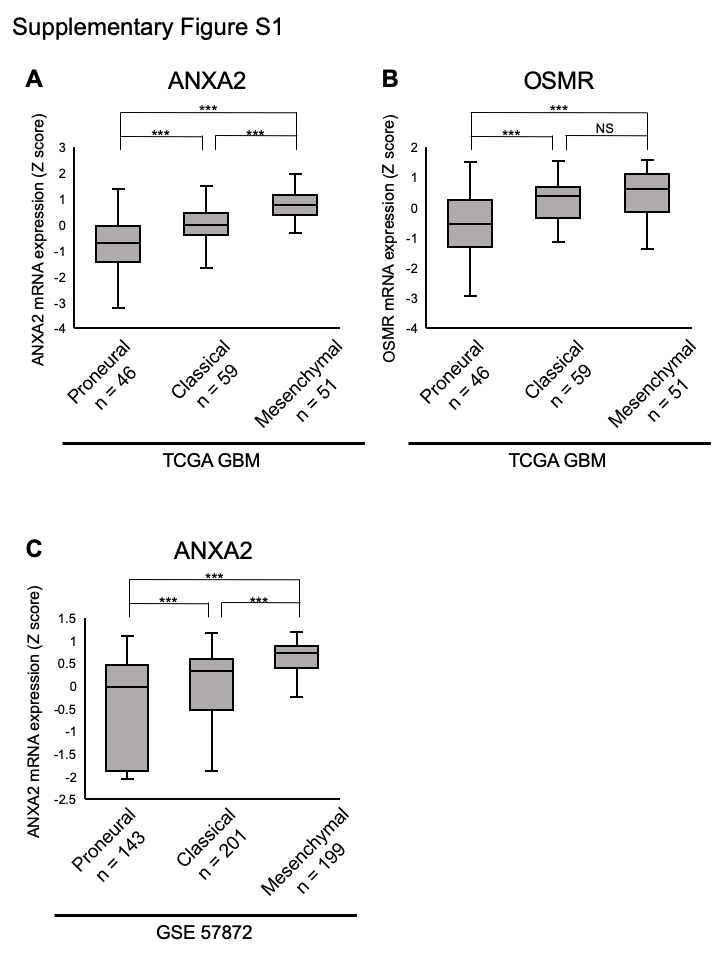

Supplement: Supplementary file 3 — Additional file 3: Supplementary Figure S1. ANXA2 and OSMR mRNA expression in the three GBM subtypes. [file 40478_2020_916_MOESM3_ESM.tiff]

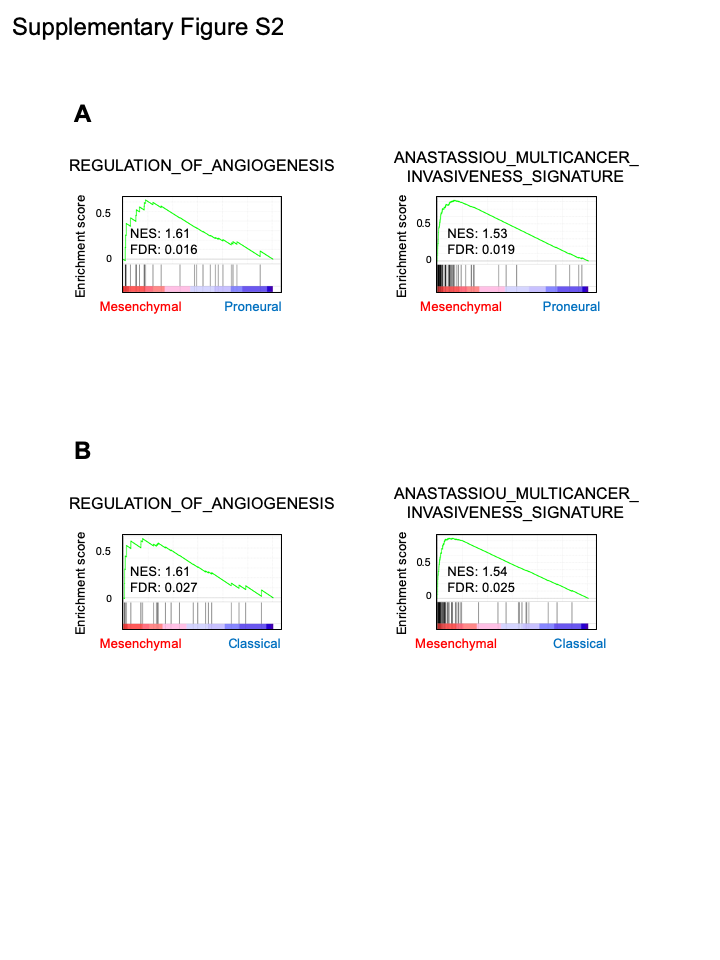

Supplement: Supplementary file 4 — Additional file 4: Supplementary Figure S2. GSEA enrichment plots of angiogenesis and invasion signatures in the four GBM subtypes. [file 40478_2020_916_MOESM4_ESM.tiff]

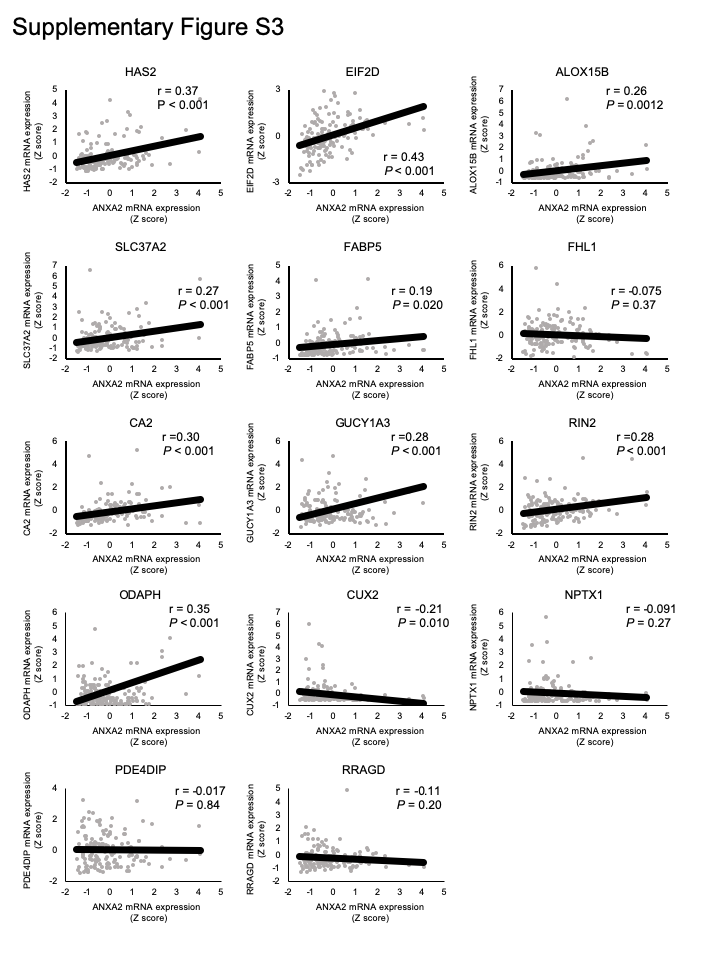

Supplement: Supplementary file 5 — Additional file 5: Supplementary Figure S3. Correlations between mRNA expression of ANXA2 and 15 genes associated with the high angiogenesis–invasion phenotype (OSMR is not shown). [file 40478_2020_916_MOESM5_ESM.tiff]

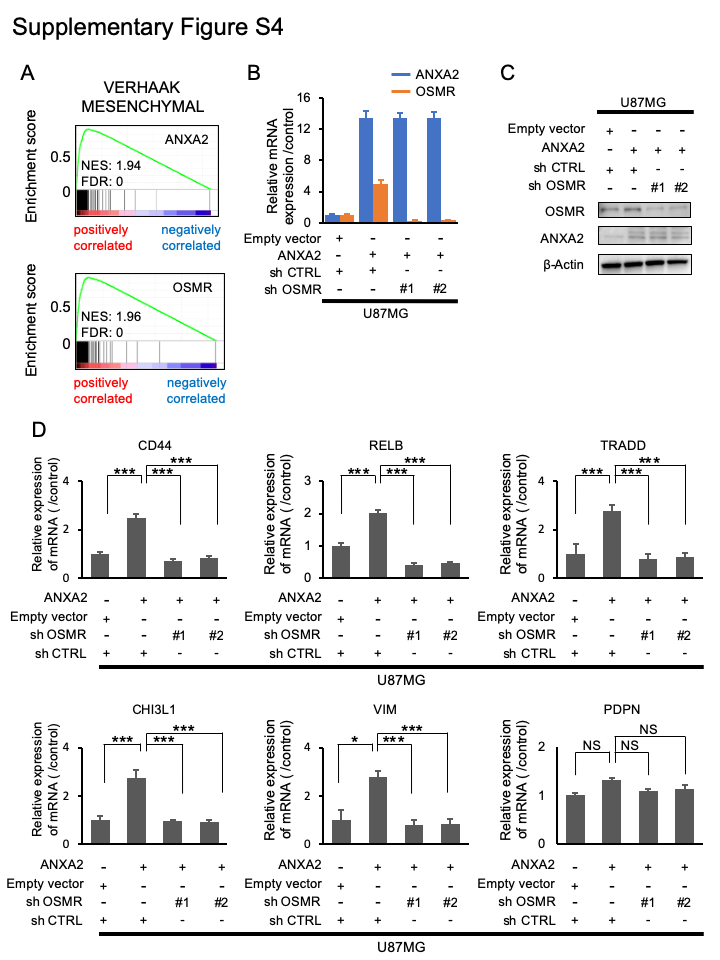

Supplement: Supplementary file 6 — Additional file 6: Supplementary Figure S4. ANXA2 and OSMR modulate the mesenchymal transition of GBM cells in vitro. [file 40478_2020_916_MOESM6_ESM.tiff]

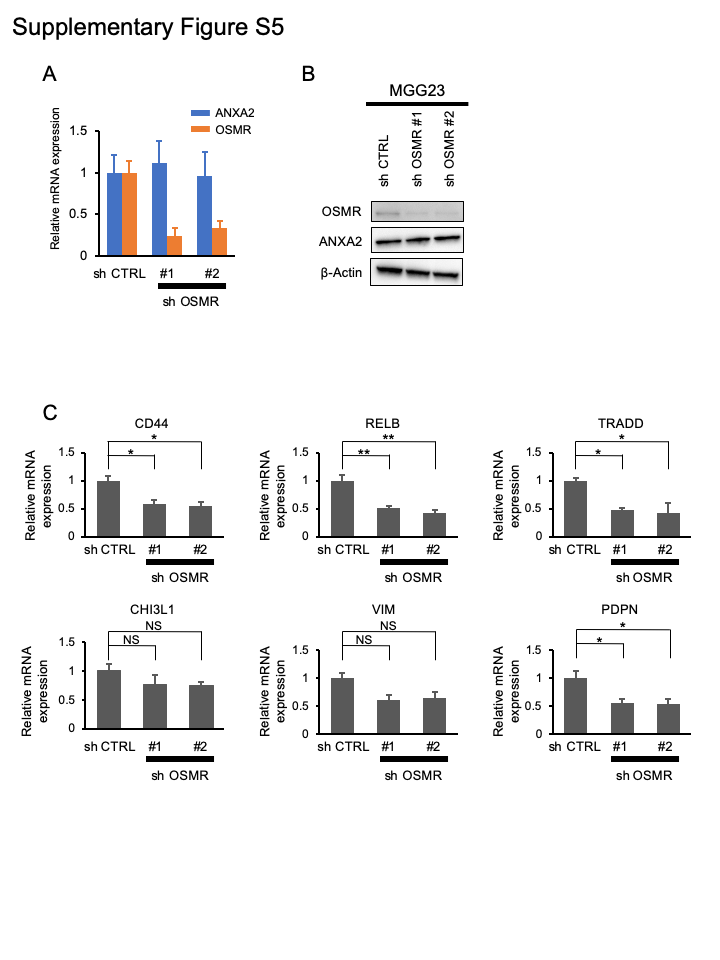

Supplement: Supplementary file 7 — Additional file 7: Supplementary Figure S5. OSMR alone modulates the mesenchymal transition of GBM cells in vitro. [file 40478_2020_916_MOESM7_ESM.tiff]

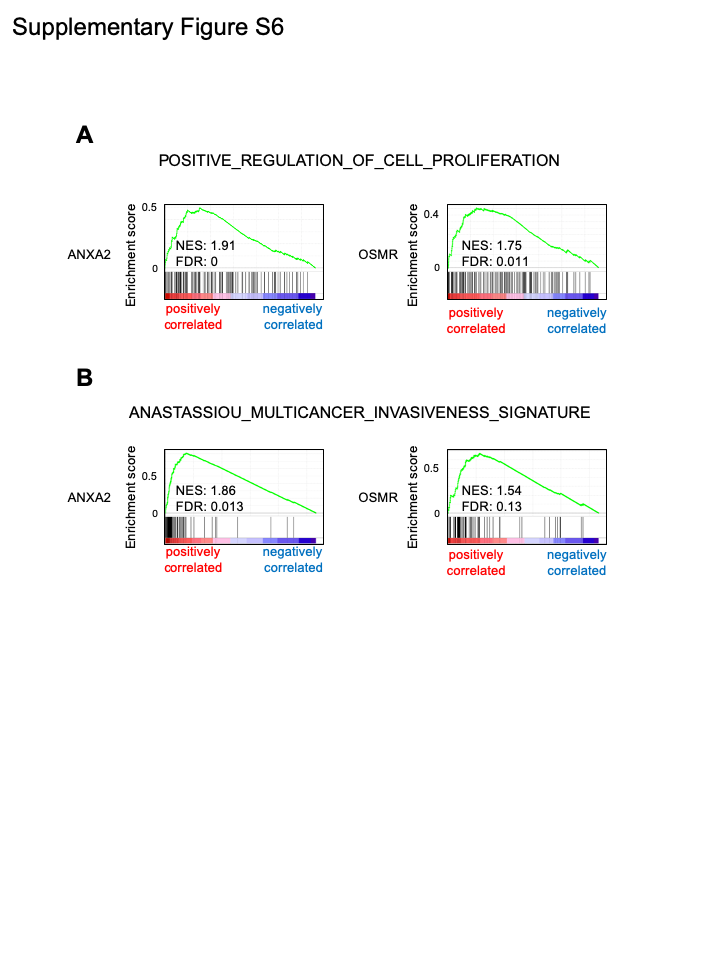

Supplement: Supplementary file 8 — Additional file 8: Supplementary Figure S6. GSEA enrichment plots of GBM patients expressing high versus low ANXA2 and OSMR mRNA levels. [file 40478_2020_916_MOESM8_ESM.tiff]

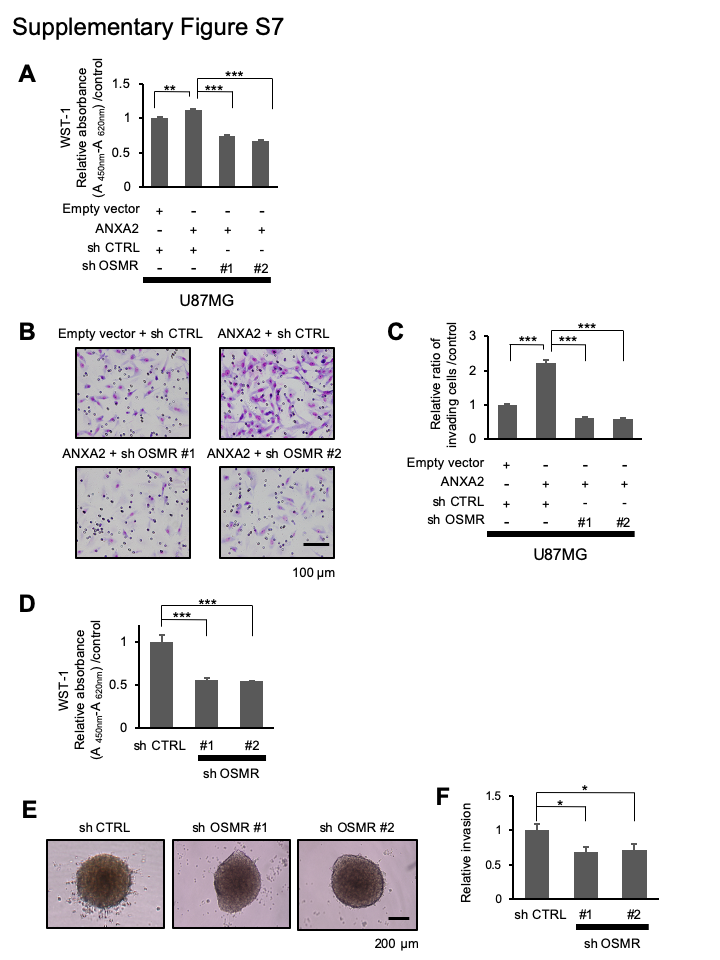

Supplement: Supplementary file 9 — Additional file 9: Supplementary Figure S7. Control of GBM cell proliferation and invasion by ANXA2 and OSMR. [file 40478_2020_916_MOESM9_ESM.tiff]

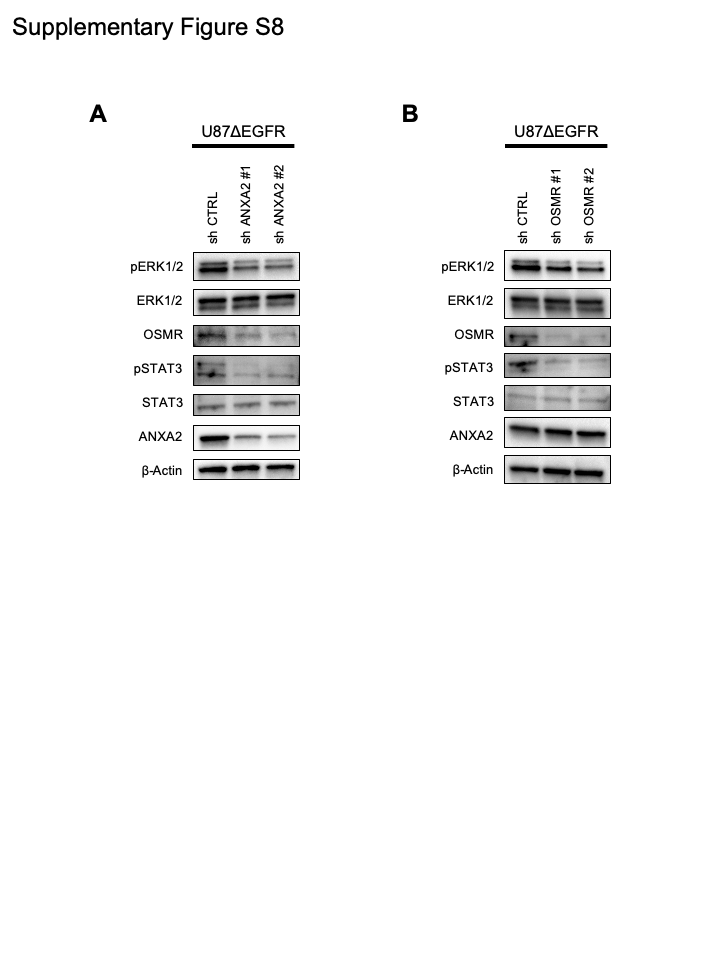

Supplement: Supplementary file 10 — Additional file 10: Supplementary Figure S8. Effect of ANXA2 and OSMR knockdown on STAT3 and ERK signaling in U87ΔEGFR cells. [file 40478_2020_916_MOESM10_ESM.tiff]

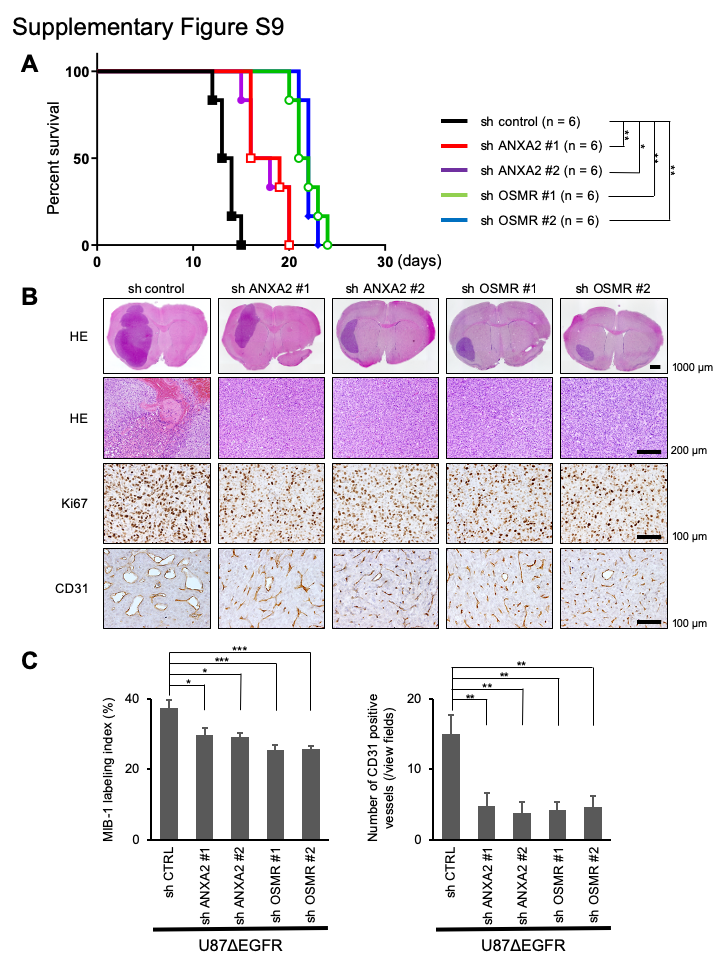

Supplement: Supplementary file 11 — Additional file 11: Supplementary Figure S9. Effect of ANXA2 and OSMR knockdown on GBM xenograft growth and phenotypic transition in mice. [file 40478_2020_916_MOESM11_ESM.tiff]

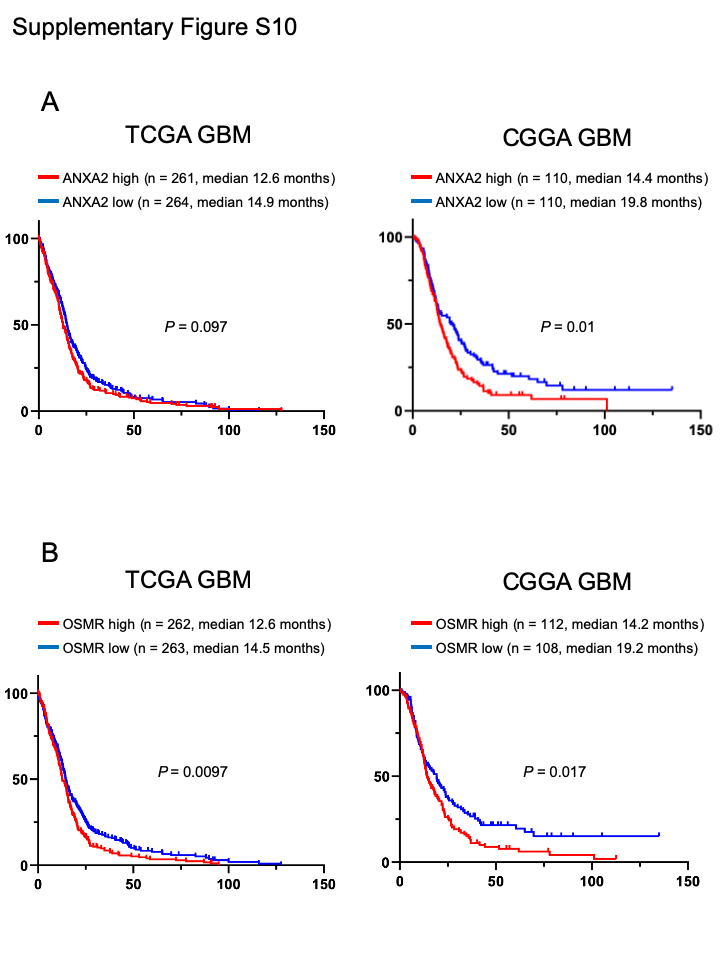

Supplement: Supplementary file 12 — Additional file 12: Supplementary Figure S10. Kaplan–Meier overall survival curves of patients in the TCGA GBM dataset and the CGGA GBM dataset stratified by high or low ANXA2 and OSMR mRNA levels. [file 40478_2020_916_MOESM12_ESM.tiff]
