# Supplementary material for: Annexin A2–STAT3–Oncostatin M receptor axis drives phenotypic and mesenchymal changes in glioblastoma
Source: Acta Neuropathol Commun. 2020 Apr 5;8:42. doi: 10.1186/s40478-020-00916-7 (PMC7132881; doi:10.1186/s40478-020-00916-7)
Supplement: Supplementary file 13 — Additional file 13. Supplementary figure legends. [file 40478_2020_916_MOESM13_ESM.docx]

Supplementary figure legends

Supplementary Figure S1. ANXA2 and OSMR mRNA expression in the three GBM subtypes.

(A, B) Box plots of ANXA2 and OSMR mRNA expression in molecular subtypes from the TCGA database (GBM subtype: proneural n = 46, classical n = 59, mesenchymal n = 51). ANXA2 expression is significantly elevated in the mesenchymal subtype compared with the remaining subtypes. OSMR expression is upregulated in the mesenchymal subtype compared with the remaining subtypes, although there are no significant differences between the classical subtype and the mesenchymal subtype. (C) Box plots of ANXA2 mRNA expression in molecular subtypes from the GSE57872 dataset (GBM subtype: proneural n = 143, classical n = 201, mesenchymal n = 199). ****P* < 0.001, NS not significant by one-way ANOVA with Bonferroni’s post hoc test.

Supplementary Figure S2. GSEA enrichment plots of angiogenesis and invasion signatures in the four GBM subtypes.

(A–C) GSEA of datasets from the TCGA database (GBM subtype: proneural n = 55, neural n = 28, classical n = 51, and mesenchymal n = 53). The mesenchymal subtype shows significant enrichment of the angiogenesis and invasion gene signature compared with the proneural (A), neural (B), and classical (C) subtypes.

Supplementary Figure S3. Correlations between mRNA expression of ANXA2 and 15 genes associated with the high angiogenesis–invasion phenotype (OSMR is not shown).

Expression data of the 15 overlapping genes in Angiogenesis-1 and Angiogenesis-2 were obtained from the TCGA dataset (n = 147). Correlations were determined using Pearson’s correlation test.

Supplementary Figure S4. ANXA2 and OSMR modulate the mesenchymal transition of GBM cells *in vitro*.

(A) GSEA of GSE4412 dataset (n = 85) showing significant enrichment of the mesenchymal gene signature in glioma patients who had elevated expression levels of ANXA2 and OSMR. (B, C) qRT-PCR (n = 3; B) and western blot (C) analysis of ANXA2 and OSMR expression in four U87MG cell lines transfected with lentiviruses encoding ANXA2 and/or an OSMR-targeting shRNA. (D) qRT-PCR analysis of mRNA expression of the mesenchymal signature genes in the U87MG cell lines described in (B, C) (n = 3). Data are shown as the mean ± SEM. **P* < 0.05, ****P* < 0.001, NS not significant by one-way ANOVA with Bonferroni’s post hoc test.

Supplementary Figure S5. OSMR alone modulates the mesenchymal transition of GBM cells in vitro.

(A) (B) qRT-PCR (n = 3; A) and western blot (B) analysis of OSMR expression in MGG23 cell lines transfected with lentiviruses encoding an OSMR-targeting shRNA. (C) qRT-PCR analysis of mRNA expression of the mesenchymal signature genes in the MGG23 cell lines described in (A, B) (n = 3). Data are shown as the mean ± SEM. **P* < 0.05, ***P* < 0.01, NS not significant by one-way ANOVA with Bonferroni’s post hoc test.

Supplementary Figure S6. GSEA enrichment plots of GBM patients expressing high *versus* low ANXA2 and OSMR mRNA levels.

GSEA plots of GSE4412 dataset (n = 85) suggest a potential role for both ANXA2 and OSMR in cell proliferation (A) and cell invasion (B).

Supplementary Figure S7. Control of GBM cell proliferation and invasion by ANXA2 and OSMR.

(A) WST-1 proliferation assay of U87MG cells transfected with lentiviruses encoding ANXA2 and/or and OSMR-targeting shRNA. Cell proliferation was measured after 2 days in culture (n = 10). (B, C) Representative images (B) and quantification (C) of Matrigel double-chamber invasion assay of U87MG cell lines described in (A). (D) WST-1 proliferation assay of MGG23 cells transfected with lentiviruses encoding an OSMR-targeting shRNA after 2 days incubation (n = 10). (E, F) Representative images (E) and quantification (F) of the Matrigel invasion assay of MGG23 cell lines described in (D). In Matrigel double-chamber invasion assay of U87MG cell lines, cell invasion was measured after a 20 h incubation (n = 4). In the Matrigel invasion assay of the MGG23 cell lines, cell invasion was measured after 10 days incubation (n = 4). Data are shown as the mean ± SEM. **P* < 0.05, ***P* < 0.01, ****P* < 0.001 by one-way ANOVA with Bonferroni’s post hoc test.

Supplementary Figure S8. Effect of ANXA2 and OSMR knockdown on STAT3 and ERK signaling in U87ΔEGFR cells.

Western blot analysis of the indicated proteins in U87ΔEGFR cells stably expressing control, ANXA2- or OSMR-targeting shRNAs. Knockdown of ANXA2 decreased OSMR expression and phosphorylation of STAT3 and ERK, whereas knockdown of OSMR reduced phosphorylation of STAT3 and ERK.

Supplementary Figure S9. Effect of ANXA2 and OSMR knockdown on GBM xenograft growth and phenotypic transition in mice.

(A–C) Kaplan–Meier survival curves of groups of BALB/c-nu/nu mice injected intracranially with U87ΔEGFR cells expressing the indicated shRNAs (n = 6). (B, C) Representative images of H&E, Ki67, and CD31 staining (B) and quantification of staining (C) in intracranial tumors excised from mice 10 days after injection of U87ΔEGFR cell lines as described in (A) (n = 5). Data are shown as the mean ± SEM. **P*<0.05, ***P* < 0.01, ****P* < 0.001 by the log-rank test (A) or one-way ANOVA with Bonferroni’s post hoc test (C).

Supplementary Figure S10. Kaplan–Meier overall survival curves of patients in the TCGA GBM dataset and the CGGA GBM dataset stratified by high or low ANXA2 (A) and OSMR (B) mRNA levels. Survival curves were compared using a log-rank test.
